# Bioavailability of Orally Administered Active Lipid Compounds from four Different Greenshell™ Mussel Formats

**DOI:** 10.3390/md18110524

**Published:** 2020-10-23

**Authors:** Matthew R. Miller, Marlena C. Kruger, Chris Wynne, Devonie Waaka, Weili Li, Chris Frampton, Fran M. Wolber, Charles Eason

**Affiliations:** 1 Cawthron Institute, 98 Halifax Street East, Nelson 7010, New Zealand; Charles.Eason@cawthron.org.nz; 2 School of Health Sciences, College of Health, Massey University, Palmerston North 4442, New Zealand; M.C.Kruger@massey.ac.nz; 3 Christchurch Clinical Studies Trust (CSST), Christchurch Central, Christchurch 8011, New Zealand; chris@ccst.co.nz (C.W.); devonie.waaka@ccst.co.nz (D.W.); 4 Department of Clinical Science and Nutrition, Faculty of Medicine, Dentistry and Life Sciences, University of Chester, Chester CH1 4BJ, UK; w.li@chester.ac.uk; 5 Department of Medicine, University of Otago, Dunedin 9016, New Zealand; chris.frampton@otago.ac.nz; 6 School of Food and Advanced Technology, College of Sciences, Massey University, Palmerston North 4442, New Zealand; F.M.Wolber@massey.ac.nz

**Keywords:** eicosapentaenoic acid (EPA), docosahexaenoic acid (DHA), green lipped mussels, *Perna canaliculus*, pharmacokinetics

## Abstract

Greenshell™ mussel (GSM, *Perna canaliculus*) is New Zealand’s most important aquaculture species. They are a good source of long chain-polyunsaturated fatty acids (*n*-3 LC PUFA). Beyond a traditional food product, GSMs are also sold as mussel powders and oil extract formats in the nutraceutical markets. In this study, a four-sequence, single dose, randomized crossover human trial with eight evaluable healthy male participants was undertaken to determine the bioavailability of the *n*-3 LC PUFA in four different GSM formats (oil, powder, food ingredient and half-shell unprocessed whole mussel) by measuring area under the curve (AUC) and maximal concentration (C_Max_). Blood samples were collected at baseline and up to 48 h after initiation of product consumption in each administration period. There were minor differences between the bioavailability of FA (fatty acid) between the different GSM formats. Eicosapentaenoic acid (EPA) peak concentrations and plasma exposures were significantly lower with GSM oil compared to GSM half-shell and GSM powder formats, which resulted in AUC_0–48_ for the intake of GSM half-shell mussel and GSM powder being significantly higher than that for GSM oil (*p* = 0.013, *f*= 4.84). This equated to a 20.6% and 24.3% increase in the amount of EPA present in the plasma after consumption of half-shell mussels and mussel powder respectively compared to GSM oil. GSM oil produced the shortest median time to maximal plasma *n*-3 LC PUFA concentration of all evaluated products demonstrated by a shorter maximum measured plasma concentration (T_Max_ = 5 h). Docosahexaenoic acid (DHA) and *n*-3 LC PUFA plasma exposure parameters were statistically comparable across the four GSM products evaluated.

## 1. Introduction

The endemic Greenshell™ mussel (GSM), *Perna canaliculus*, is New Zealand’s most valuable aquaculture species in terms of both total value and scale of production. In 2019, the New Zealand GSM industry produced export revenue of NZ$337 M from 30,551 tons of exported product ^1^. The majority of GSM export sales (>80%) are presently sold as frozen half-shell food products. However, there has been recent significant growth in nutraceutical powder and oil product production, with sales of NZ$45.7 M in 2018 equivalent to a 334% increase in the last 8 years. These nutraceuticals command high prices, with oil extracts selling for ca. NZ$1900 per kg (in 2018) as opposed to freeze-dried mussel powders ca. NZ$45 per kg. Furthermore, the industry is actively working to develop novel food ingredients for use in a variety of different food formats, to reach markets where live and frozen GSM cannot penetrate.

To the best of our knowledge, GSM oil is the most valuable marine oil (by price) in the world, sitting presently at over $1300 USD/kg [1]. The GSM lipid fraction contains a high proportion of omega-3 long-chain (C ≥ 20) polyunsaturated fatty acids (*n*-3 LC PUFAs), predominantly (DHA, 22:6 *n*-3) and eicosapentaenoic acid (EPA, 20:5 *n*-3), which are split between the triacyglycerol (TAG) and polar lipid (PL) classes [2,3]. These GSM products have been extensively tested in pre-clinical animal studies [4,5]. A recent review has identified a gap in the literature for GSM active ingredients regarding their pharmacokinetics and identification of the bioactive factors responsible for each observed therapeutic effect [4].

The health benefits of *n*-3 LC PUFA, such as DHA and EPA, have been extensively studied and found to include anti-inflammatory activity, maintenance of normal heart function and lipid profile, and hypertriglyceridemia management [6,7,8,9]. Due to these health benefits, *n*-3 LC PUFA enriched materials have been widely used in functional foods and nutritional supplements [10,11,12]. European Food Safety Authority (2011) approved the health claims of (DHA) in maintaining the normal version and normal heart function with consumption of 250 mg/day and maintaining cardiac function with consumption of EPA and DHA 250 mg /day [13]. More recently, Abort et al., (2020) investigated the anti-inflammatory bioactivity of intake of DHA-enriched fish oil against type 2 diabetes (T2D) development [14]. The results showed a significant reduction in Insulin Resistance (IR) in adults with abdominal obesity through a double-blinded, randomized, parallel-arm placebo-controlled trial.

In addition, more research evidence showed that normal function of the brain with relation to DHA was related to its bound structure and doses of intake. Sugasini et al., (2019) studied the effects of dietary DHA of TAG, PC (Phosphatidylcholine) and LPC (Lysophosphatidylcholine) on enrichment of brain DHA and found that that DHA of TAG and PC (sn-2 position) were not suitable for brain enrichment, but sn-1 and sn-2 of LPC and sn-1 PC were functionally effective [15]. Arellanes et al. (2020) also demonstrated that DHA sn3-FA (2 g/day) or higher TAG-DHA were needed to ensure adequate delivery of DHA to brain for those in the high risk of dementia group who are APOE4 (apolipoprotein E4) carriers [16]. Interestingly, Das (2020) proposed that that administration of anti-inflammatory bioactive lipids, especially arachidonic acid, could be of significant benefit in prevention and management of COVID-19 (coronavirus disease of 2019) and other enveloped viruses [17].

However, there are challenges in evaluating efficacy of *n*-3 LC PUFA in delivering health benefits [7] as the digestion of lipophilic components of foods and nutritional supplements is subject to a complex series of processes including bile salt emulsification and pancreatic lipase hydrolysis [18]. Account must be taken of the considerable variability in the absorption of different *n*-3 LC PUFAs, as well as the format they are in (i.e., lipid class) which may have a direct impact on their bioactivity [12,19].

Nutraceutical products from GSM (both oil and powders) have been shown to have benefits in human clinical trials on the alleviation of osteo-/rheumatoid-arthritis [20,21,22], asthma [23,24], and adverse effects of exercise and/or inflammation [25,26]. With some new animal studies showing novel activity in protecting against cartilage damage in both early-stage and late-stage metabolic osteoarthritis [5], increasing lean mass accrual in diet-driven obesity, and reducing loss of bone mineral density in osteoporosis [27]. Due to the low lipid content of mussels (~2%), nutraceutical oils from GSM are produced on an industrial scale either by solvent extraction or by supercritical CO_2_ extraction. Freeze-and spray-drying are the most commonly utilized methods for producing GSM powders, which are utilized as nutraceuticals and are being developed as food ingredients. It has been identified that in these GSM nutraceutical studies using animal and clinical models, there were inconsistencies in the degree of benefit provided, which could be attributed to the different dosages used and the different types of GSM extracts used [4]. In turn, these differences could affect the concentrations of key bioactives present.

The bioavailability and pharmacokinetics (PK) of a drug are key determinant factors in the ultimate manifestation of efficacy in clinical practice. The same applies to bioactives from natural sources that deliver health benefits such as *n*-3 LC PUFA. Critically important parameters are the C_Max_ (Maximum measured plasma concentration from time), T_Max_ (Time of the maximum measured plasma concentration), elimination t^½^ and AUC (incremental area under the plasma concentration-time curve) data after single and multiple doses, which are used to determine optimum dosing regimens. Various biomarkers (such as EPA and DHA) have been used in recent studies on the bioavailability of *n*-3 LC PUFA, but their effectiveness has not been systematically compared nor has their use been fully justified [10,11,19,28,29,30]. In addition, there is growing evidence that the bioavailability of *n*-3 LC PUFAs varies depending upon their derived sources, such as krill oil, fish oil, mussel or algal oils [10,11,12], and is further dependent on the fat composition and content of the diet and the food preparation methods employed [10,31]. This is due to the food structure or format of the *n*-3 LC PUFAs of the difference sources. The difference in the bioavailability of *n*-3 LC PUFAs when eaten as food product, functional food, freeze dried powder and/or extracted oil is yet to be established and we hypothesize that it may differ as food structure and therefore digestion may be affected by format. In this paper, pharmacokinetic parameters to determine bioavailability (C_Max_, T_Max_ and AUC) of *n*-3 LC PUFA were measured for different formats of GSM following single dose intakes, as a first step in gaining a better understanding of the pharmacokinetics of key active ingredients.

There are 40 years of scientific literature connecting GSM extracts with health benefits; however, there are major knowledge gaps preventing validated health claims, particularly around consistent efficacy and proven bioavailability. This study was intended to evaluate comparative pharmacokinetics and bioavailability of orally administered bioactive compounds from whole mussels, nutraceutical products and a novel functional food ingredient. A four-sequence, single dose, randomized crossover study in healthy adults was undertaken to determine the bioavailability of *n*-3 LC PUFAs, EPA and DHA in different GSM formats (frozen half-shell GSM, GSM food ingredient, GSM powder and GSM oil extract). Blood samples were collected pre-dose and up to 48 h with 2 h intervals in the initial 8 h and 4–8 h intervals in the later stage after initiation of product administration. The aim of this study was to assess the uptake and retention of the *n*-3 LC PUFA by healthy adults from the four different formats.

## 2. Results

A total of twelve subjects were screened for the study. One subject failed screening based on an exclusionary BMI. Two further subjects met eligibility criteria at screening but subsequently withdrew consent prior to study enrollment.

Nine subjects were randomized into the study and were included in the safety population. This included one subject (subject 04) who withdrew consent prior to completing PK sampling for Administration Period 1; a replacement subject (subject 14) was randomized into the study, to ensure eight evaluable subjects.

All subjects in the safety analysis set identified their ethnicity as European (7 New Zealand European, 1 Spanish, 1 European). The mean age of subjects was 23 years (range 20–30 years), with mean BMI 24.4 kg/m^2^ (range 21.5–30.7 kg/m^2^).

**Figure 1:**
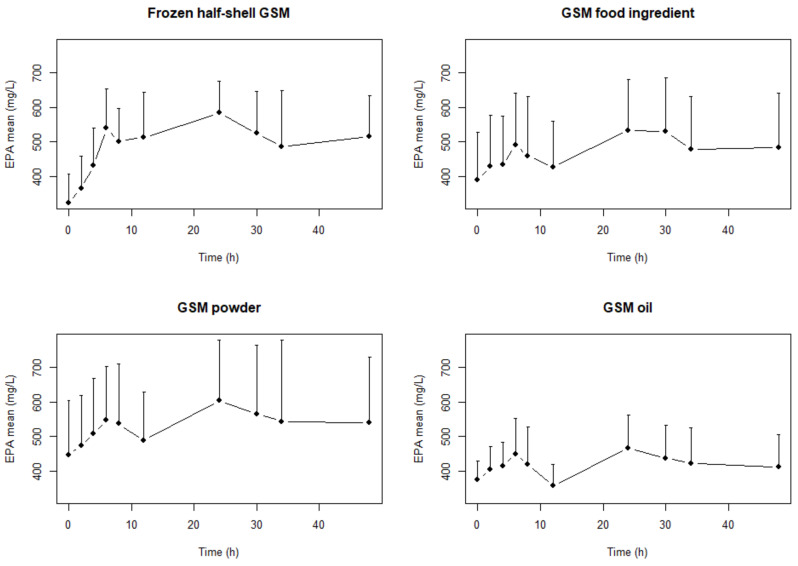
Mean (+SD) eicosapentaenoic acid (EPA) concentration–time profiles (baseline-adjusted change) after a single dose of frozen half-shell GSM, GSM food ingredient, GSM powder and GSM oil extract with matching levels of lipid.

**Figure 2:**
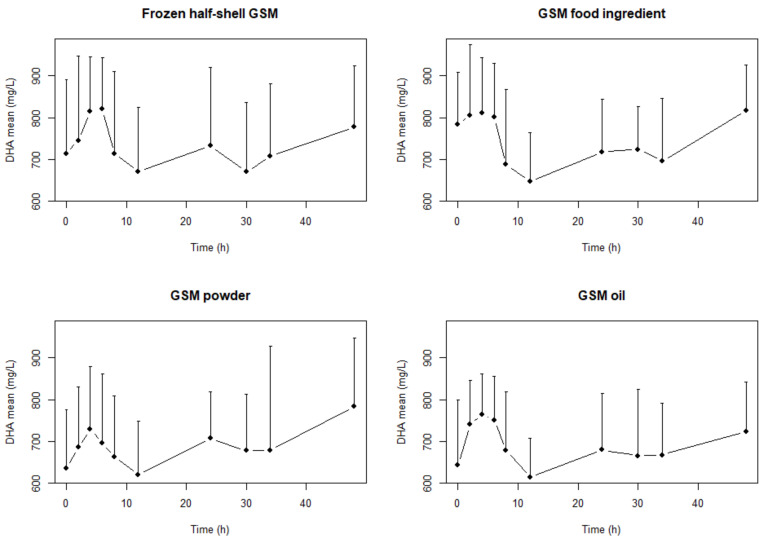
Mean (+SD) docosahexaenoic acid (DHA) concentration–time profiles (baseline-adjusted change) after a single dose of frozen half-shell GSM, GSM food ingredient, GSM powder and GSM oil extract with matching levels of lipid.

**Figure 3:**
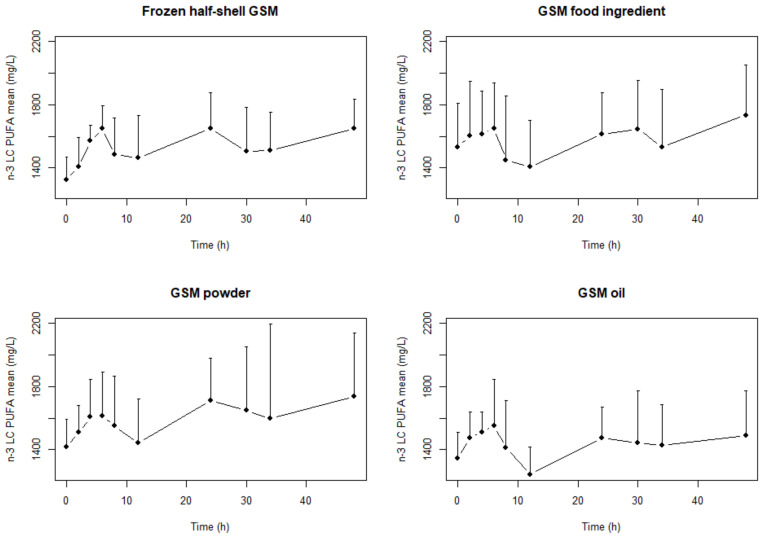
Mean (+SD) *n*-3 LC PUFA (>18 carbon length) concentration–time profiles after a single dose of frozen half-shell GSM, GSM food ingredient, GSM powder and GSM oil extract with matching levels of lipid.

The mean plasma concentrations of EPA, DHA and total *n*-3 PUFA (all omega 3 fatty acids including α-linolenic acid (ALA, 18:3 *n*-3), stearidonic acid (SDA, 18:4 *n*-3), eicosatetraenoic acid (ETA 20:4 *n*-3), EPA, docosapentaenoic acid (DPA,22:5 *n*-3) and DHA) after a single dose of the four GSM products are shown in Figure 1, Figure 2 and Figure 3, respectively. Two distinct periods identified and analyzed for AUC, C_Max_ and T_Max_ which were the periods between 0–12 h and the whole period (0–48 h) (Table 1, Table 2 and Table 3). The values in Table 1, Table 2 and Table 3 are calculated from the raw plasma concentrations which are not corrected for variations in the baseline values. Figure 1, Figure 2 and Figure 3 presents baseline adjusted data.

There were significantly (*p* = 0.013, *f* = 4.84) higher AUC in EPA concentrations with the GSM half-shell mussel and GSM powder compared to the GSM oil over the 48-h period (Table 1). However, there were no differences in the EPA in the shorter 0–12 h range. In the DHA and the *n*-3 LC PUFA there were no statistical differences between treatments in the AUC over either of the different time periods.

The C_Max0–12h_ and C_Max0–48h_ were significantly (*p* = 0.011, *f* = 5.05 and *p* = 0.004, *f* = 6.39, respectively) higher in the EPA of the GSM half-shell compared with GSM food ingredient and GSM oil over both time periods. Further, the C_Max0–12h_ and C_Max0–48h_ of the GSM powder were significantly different (*p* = 0.011, *f* = 5.05 and *p* = 0.004, *f* = 6.39, respectively) between the GSM powder and the GSM oil. italic.

The T_Max0–48h_ was significantly (*p* = 0.04) lower (5 h) in the GSM oil than the other three formats (in the 24–27 range) but this was not evident in the shorter T_Max0–12h_ period. Two peaks are identified (Figure 1, Figure 2 and Figure 3) in the concentration-time profiles which led to two time periods analyzed for maximum measured plasma concentration (0–12 h and 12–48 h). There were no statistical differences determined in the T_Max0–12h_ and T_Max0–48h_ in EPA and DHA concentrations during the study.

All evaluated GSM food platforms were well tolerated. A total of three subjects (33.3%) experienced a total of three adverse events (AEs). Two AEs (dry throat and upper respiratory tract infection) were considered unrelated to test product; the remaining AE of a headache was considered by the Investigator to have a remote relationship to test product. The three reported AEs were graded as mild in severity, required no specific intervention and resolved spontaneously. No deaths, serious AEs or AEs resulting in discontinuation of study product were reported.

## 3. Discussion

In this study, we measured the pharmacokinetic parameters (AUC, C_Max_ and T_Max_) for the bioactive *n*-3 LC PUFA in human subjects fed different forms of GSM. Two time periods were analyzed as there were two distinct peaks in the bioavailability of these bioactive lipids (Figure 1, Figure 2 and Figure 3). These two distinct periods have been previously reported in a high PL *n*-3 LC PUFA bioavailability study [32]. The whole 48 h period pharmacokinetic parameters were determined as well as the shorter 0–12 h (Table 1, Table 2 and Table 3). These two time periods allowed for good comparison with published literature results (most of which were over the 0–12 h period) and also extrapolation with longer-term studies. This research addresses the essential step in understanding the bioavailability of key bioactives (*n*-3 LC PUFA) in GSM formats. The lack of bioavailability data in the literature for GSM, in both animal and clinical models, has enhanced inconsistencies with the efficacy data and makes comparisons between trials/formats difficult.

The results showed differences only in the bioavailability of EPA in the GSM half-shell mussel and GSM powder compared to the GSM oil over the 48-h period with significant (*p* = 0.013, *f* = 4.84) differences in the AUC_0–48t_ (Table 1). This corresponds to a 20.6% and 24.3% increase in the amount of EPA present in the plasma after consumption of half-shell mussels and mussel powder respectively. The GSM food ingredient had a similar bioavailability of EPA to the GSM half-shell and GSM powder products but was not significantly different to that of the GSM oil. In the DHA and *n*-3 LC PUFA, there were no differences determined between the four different GSM formats.

The C_Max_ of EPA was significantly lower for the GSM oil than the GSM half-shell and GSM powder formats in both the 0–12 h and 0–48 h time frames (Table 2). Further there was a significant difference in C_Max_ between the GSM half-shell mussels and the GSM food ingredient for both time periods. This relates to a 22–25% increase in EPA in the plasma if delivered as a GSM half-shell and GSM powder compared to extracted GSM oil depending on the timeframe calculated. The extracted oil (GSM oil) had a significantly shorter time (5 h) to reach the time of the maximum measured plasma concentration T_Max_ of *n*-3 LC PUFA than the other three formats, which were between 24–30 h only when assessed over the 48 h period (Table 3).

The data (shown in the tables) were not baseline-adjusted due to the fact that many of the adjusted AUCs were negative for all formats and therefore it was not possible to analyze these with the transformed ANOVA models. However, the figures were all adjusted to account for differences in baseline levels of omega 3. Further we intended to calculate half-life (t^½^) of the *n*-3 LC PUFA but the plasma levels did not equalize to baseline in 48h and stayed high for the duration of the study, and therefore the calculation was not possible. The t^½^ is an important kinetic parameter and is worthy of mention due to the longevity of the key actives in the plasma, which could indicate one or two feeds a week would provide elevated levels of *n*-3 LC PUFA.

Different food formats will deliver *n*-3 LC PUFA in different molecular forms. In this study, we attempted to match the lipid content (2.2 ± 0.1 g/100 g) across the four formats with only the food ingredient (2.05 g/100 g) not meeting that requirement (Table 4). However, the lipid classes and the amount of *n*-3 LC PUFA were different. The GSM oil and frozen half-shell mussels were predominately in the form of PL (64% and 68%, respectively), whilst the food ingredient and the mussel powder were mainly TAG (97% and 94%, respectively). EPA was consistent across the four diets (341 ± 15 mg/100 g); however, the DHA content was markedly different. The frozen half-shell mussels had the highest content of DHA (311 mg/100 g) followed by the oil (267 mg/100 g), then the two powders (174 and 189 mg/100 g for the food ingredient and mussel powder, respectively). These differences in content contributes uncertainty to our results but using natural and food products as a part of a clinical trials it is hard to get interventions that are uniform in composition due to natural variation in these products.

The GSM oil and particularly the GSM half-shell mussel formats were abundant in these phospholipids (identified in this paper as PL), but there has yet to be a lipidomic profile determining the phospholipid profile of GSM published. PL has the capability to act as an emulsifier, enhancing the formation of lipid extract emulsion in the digestion. In general, in marine oils the more unsaturated fatty acids are most likely to attach to the sn2-position. It has been shown in GSM that EPA is more abundant in the TAG fraction than the PL fraction when extracted chemically (the opposite was shown in supercritical extracted fraction albeit the PL fraction was a very minor part of that oil) [2]. However, there was no statistical difference in the concentration of DHA across the non-polar and polar fractions [2]. The PL fraction has been reported to be the predominant lipid across 12 months of sampling in the GSM production areas, and *n*-3 PUFAs were consistent across the seasons with 35–38% of all fatty acids primarily made up of EPA and DHA [3]. Further analysis has shown that there are differences in the concentrations of EPA (but not DHA) dependent on gender (with females having 43% more EPA than males) as well as significant differences in the lipid classes (with PL lower and TAG greater in female mussels) [33]. In this study, we only used female mussels to reduce the impact of these gender differences. Both the GSM powder and GSM food ingredient have been processed to form these products (e.g., homogenizing, drying, etc.) and through this process the PL fraction of the lipid profile was minor (see Table 4). Therefore, the bioavailability of EPA and DHA in GSM, independent of format, may be affected by factors such as season, extraction method, processing method and gender of mussels due to structural and concentration changes of the bioactive FA and these may account for some of the differences we have shown in this study as well as discrepancies in the published literature.

There have been minimal and conflicting studies comparing the bioavailability of EPA and DHA when delivered in different food formats, e.g., oil in a capsule verses fish as a meal. Visioli et al. [34] showed increased levels of plasma EPA and DHA when they fed salmon compared to capsules but attributed their finding to the larger fat content of the meal. They concluded that fish is more effective than capsules in providing *n*-3 LC PUFAs. Elvevoll et al. [35] showed fish consumption is more effective in increasing serum EPA and DHA than supplementing the diet with fish oil. In contrast, Stonehouse et al. [36] showed salmon oil or fillets were equally effective in delivering *n*-3 LC PUFA; however, there was additional benefit of increased levels of plasma selenium in the healthy volunteers who ate the fish. Finally, Harris et al. [37] determined there was no difference between oily fish (2 servings of oily fish e.g., salmon and albacore tuna per week) and fish-oil (1–2 capsules per day) on enriching blood lipids with *n*-3 FA.

Our GSM formats were all consumed with a low-fat soup meal (see Section 4.1) to reduce influence of breakfast/food has on the intake of *n*-3 LC PUFA. It has been shown that the lipid content of a meal in postprandial studies such as a high fat breakfast can influence plasma omega 3 levels [18]. Diet was restricted of n-3 LC PUFA and standardized over during the clinic visits with further diet, medication and lifestyle restrictions that applied over the duration of the study (see Section 4.3 for details).

Our results show that there is a slight but significant GSM format-dependent increase in AUC0–48 h, CMax and TMax0–48 h for EPA but not DHA or n-3 LC PUFAs. Our results do not conclusively answer the differences in food formats on the bioavailability of EPA and DHA and acknowledge the format of the EPA (whether on the position on the glycerol backbone or in the lipid class) may play a vital role in its bioavailability. We provided the food in a form of a soup to reduce the effect of a large fat content of the meal as a pervious study [34] indicated this may be an confounding factor. However, due to the nature of all the food products delivering *n*-3 LC PUFA through different lipid classes (e.g., TAG or PL), metabolic fates would be different, and comparisons are not identical.

There are limitations of the trial which are worthy of discussion. Firstly, participants were not screened for intake of *n*-3 LC PUFA prior to the trial and therefore bioavailability results may be different in individuals who have a high baseline *n*-3 LC PUFA status. Sample size is also a limitation, however this is an exploratory study and the small sample size is sufficient to assess these fatty acid pharmacokinetic parameters. Future studies may want to consider a longer wash out period between administration and a study length of greater than 48 h. A two week wash out would be preferable as some LC-PUFA, particularly as DHA concentrations appear to be still increasing at 48 h and the 14-day wash will achieve a better chance to return to baseline. The study was limited to male participants, to reduce effect of different *n*-3 LC PUFA biosynthetic capacity between genders [38] and may not extrapolate to females. A final limitation of this trial was a complete nutritional record (24-h food recall and/or food frequency questionnaire (FFQ)) for intake of *n*-3 LC PUFA was not undertaken prior or during the trial.

Reviews of short-term post-prandial studies have reported that the type of lipid has implications on the bioavailability [18,39]. The bioavailability of EPA and DHA in the ethyl ester form (EE, a synthetic form commonly used for nutraceutical lipids and particularly used in concentrated delivery systems) were significantly lower than in TAG [18,34]. Further complexity occurs when comparing PL and TAG forms, as the literature suggests that these lipid classes are metabolized via different mechanisms and possibly have different fates. It has been shown that labeled ^13^C DHA in the PC form has markedly different kinetics and metabolic fate compared to TAG [40]. A recent review of human clinical trials found no conclusive evidence that the bioavailability of *n*-3 LC PUFA was greater from PL versus TAG sources; however, there was some evidence of increase bioavailability of *n*-3 LC PUFA delivered as PL in animal models 39.

In this trial, the different delivery formats had differing proportions of TAG and PL. Both the GSM oil (94%) and GSM half-shell (68%) were predominantly PL, whereas the opposite was true for the GSM powder (92% TAG) and GSM food ingredient (95% TAG). However, the differences we demonstrated in the EPA in AUC, T_Max_ and C_Max_ were seen between the GSM oil and GSM half-shell, which had similar lipid class profiles. Further, the GSM powder and GSM oil, which have opposite lipid class profiles, had significantly different pharmacokinetic profiles in regard to EPA. This evidence suggests that lipid class can play a role in the bioavailability of FA from GSM.

Experimental and human studies with GSM now span some 40 years, and although animal veterinary and clinical trials have been conducted in the last 15 years there is still considerable inter-study variation in the ingredients and dosages used. Sound quality assurance (QA) on raw materials and formulations of GSM ingredients have been lacking as well as poor understanding of absorption, bioavailability, distribution, metabolism and excretion of key active ingredients. As our understanding on the key active ingredients in GSM extracts improves, analytical methodology can be used to assess the concentration of these bioactives in GSM foods, extracts and final product. This will open up new research avenues as more pharmacokinetic studies become a possibility. It will also allow GSM breeding programs to select for mussel families that produce higher levels of the bioactives of interest, with good bioavailability yielding a more potent feedstock for therapeutic usage in animals and humans alike. This single dose trial has provided early insights which will need to be complemented by multi-dose trials in clinical settings to further establish the bioavailability and pharmacokinetics of GSM bioactive components.

In summary, the AUC, C_Max_ and T_Max_ of EPA were affected by the delivery format. This may be related to EPA’s positions on the glycerol backbone as well as the processing procedures. The evidence suggests that lipid class (PL or TAG format) plays a role in the bioavailability of EPA. EPA has previously been shown to be more bioavailable in terms of T_Max_ than DHA [12]. T_Max_ for EPA and DHA is in the range 2 h–24 h, which may be related not only to their molecular chains and bonding structure, but also to the format by which they were delivered. In our study we were able to show there were no differences in DHA or *n*-3 LC PUFA in terms of AUC, C_Max_ and T_Max_ in any of the four GSM formats.

## 4. Materials and Methods

### 4.1. GSM Food Formats

Four food formats were prepared to match in lipid content (Table 4):

1. Frozen half-shell GSM—125 g Sanford (Blanched GSM, Orvida brand, Sanford, Havelock NZ, Lot # M177812907, production date 9 May 2018);
2. GSM food ingredient—22.5 g Sanford (Batch L001, production 25 March 2018);
3. GSM powder—22.5 g Enzaq (Batch L457–2, production 8 July 2018);
4. GSM oil extract—2.3 g Pernatec oil (Waitaki Bioscience, production date 5 May 2018).

All samples were aliquoted and kept frozen until administration during the trial period of 31 July–24 September 2018. The food ingredient is an activated GSM powder product developed to be utilized in the incorporation of functional foods.

To control for the effects of whole-meal components, all four GSM products were served in the same format, a leek and potato soup (204.3 g per portion) comprised of the ingredients; 600 g Leeks, 250, Onions, 5 g Garlic, 30 g unsalted butter, 350 g Potato (agria), 600 g Chicken stock, 2 g Bay leaf, 0.2 g Thyme, 1 tsp Iodized salt, Ground black pepper and 200 mL low fat milk. The study was conducted from 31 July–24 September 2018 at the Christchurch Clinical Studies Trust Ltd. (CSST) site in Christchurch, New Zealand. The study was approved from the Central Health and Disability Ethics Committee (18/CEN/120) and conducted in accordance with ICH GCP and local ethical and regulatory requirements.

### 4.2. Clinical Study

The study was designed as an open-label, randomized, four-sequence crossover trial in eight evaluable healthy adult male participants. Administration occurred over the course of four administration periods, with a wash-out of at least seven days between test products.

The open-label, randomized cross-over design was utilized to enable both intra-subject and inter-subject comparison. No formal sample size calculations were made for this study, however the sample size of eight was expected to be adequate for evaluation of the parameters of interest. Single exposure pharmacokinetics allowed adequate characterization of the performance of each product and was therefore appropriate in meeting the study objective.

The dose of each product was selected to have similar lipid content (2.2 ± 0.1 g). Products were tested by the accredited Cawthron food testing laboratory, to determine accurate lipid and fatty acid content. The one-week washout interval utilized in the current trial was based on a previous study [41].

Key study inclusion criteria limited the study to healthy adult males aged 18–45 years, body mass index (BMI) 18 to 32 kg/m^2^ inclusive, with no clinically significant medical conditions. The study population was selected to reduce inter-subject variability and is considered appropriate for a non-therapeutic trial assessing pharmacokinetic parameters.

All subjects were provided with written and oral information about the study prior to the screening visit. Subjects were required to sign and date the most current Independent Ethics Committee (IEC)-approved written informed consent form before any study specific assessments or procedures were performed.

Potential subjects underwent screening procedures up to 28 days before the Day 1 of the first assessment period, to evaluate their eligibility for the study. Demographics, relevant medical history and medication history were recorded. Subjects underwent a complete physical examination. Body weight, height (and derived BMI), vital signs and a single 12-lead electrocardiogram (ECG) were recorded. Blood and urine samples were collected for laboratory safety tests (hematology, biochemistry, urinalysis). A urine drug screen and breath alcohol tests were performed.

**Figure 4:**
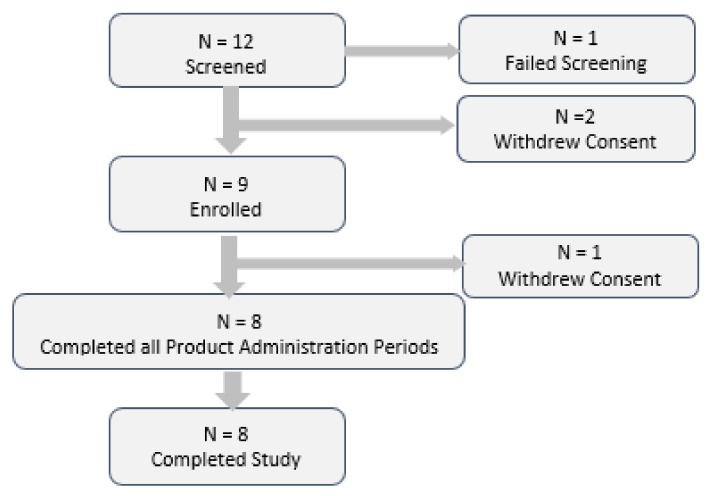
Disposition of study subjects.

Following screening procedures, eligible subjects were enrolled into the study and completed four administration periods at the CCST research unit. A total of twelve subjects were screened for the study. One subject failed screening based on an exclusionary BMI. Two further subjects met eligibility criteria at screening but subsequently withdrew consent prior to study enrolment (Figure 4). For each subject, product administration order was determined by randomized allocation to one of four Test Product sequences. Nine subjects were randomized into the study. This included one subject who withdrew consent prior to Administration Period 2; a replacement subject was randomized into the study, to ensure eight evaluable subjects.

Each administration period comprised a long clinic visit (from approximately 1½ h prior to product administration through until completion of 12-h post-administration assessments) and several short visits.

During each Administration Period PK samples were collected pre-dose and 2, 4, 6, 8, 12, 24, 30, 34 and 48 h after initiation of product administration, for measurement of plasma EPA, DHA and *n*-3 LC PUFA. Blood samples were centrifuged at 4000 rpm (1520 g) at 4 °C for 10 min and the plasma was taken and stored at −80 °C, cryopacked and shipped to Cawthron Institute for analysis.

**Figure 5:**
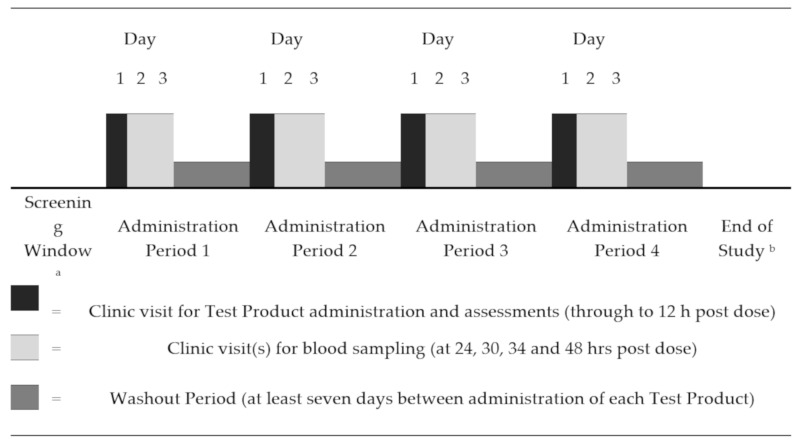
Study Design: ^a^ Up to 28 Days prior to Day 1 of Administration Period 1; ^b^ On satisfactory completion of all Administration Period 4 procedures.

Subjects were discharged from the study on completion of the final administration period assessments. Total study duration for each subject was up to approximately 90 days. Figure 5 gives an overview of the study design.

### 4.3. Protocol Restrictions

Dietary, medication and lifestyle restrictions were applied during the study. At each visit subjects were reminded of the study restrictions and each subject’s compliance was assessed. Restrictions were as follows:

- No prescription or over-the-counter medications, vitamins, minerals, or herbal supplements were permitted, from 2 weeks prior to first test product administration until study completion. Although medications required to treat adverse events were permissible with approval from the Investigator, no concomitant medications were used by any subjects during the study.
- Consumption of fish or other seafoods, or fish/seafood-containing products was not permitted, from two weeks prior to first test product administration until study completion.
- A fat-controlled diet was required, from 1 week prior to first test product administration until study completion.

Standardized meals were provided during long clinic visits, with no other food permitted.

- Alcohol was not permitted, from 48 h prior to each test product administration until the final PK sample collection in each administration period. A negative alcohol breath test was required at screening and prior to test product administration.
- Consumption of cigarettes or other nicotine-containing products was not permitted, from screening until study completion.
- Recreational drug use was not permitted, from screening until study completion. A negative urine drugs of abuse screen was a requirement for study entry.
- Water was not permitted, for one hour pre- and post-test product administration. Water was otherwise permitted ad libitum.

### 4.4. Assessment of Safety

Clinical laboratory tests and electrocardiograms were performed at screening only, to confirm study eligibility. Tolerability during the study was assessed by monitoring adverse events (AEs) and vital signs (recorded pre-dose and 12-h post-dose in each Administration Period, and prior to discharge from the study).

### 4.5. Lipid Analysis

Plasma was defrosted (500 µL aliquot) and 250 µL cold isotonic saline (0.9%) and 1 mL isopropanol (containing 0.005% Butylated hydroxyanisole (BHA) as an antioxidant) were added and vortexed thoroughly. Chloroform (2 mL, containing 0.005% BHA as an antioxidant) was added and mixed thoroughly for 10 min. The samples were centrifuged (Eppendorf 5810R, Hamburg, Germany) at 3000 rpm (850 *g*) at 4 °C for 10 min. The bottom organic phase was sampled and concentrated under nitrogen prior to methylation. The sample was trans-methylated in methanol: chloroform: hydrochloric acid (10:1:1, *v/v/v*) for 1 h at 100 °C. After addition of water the mixture was extracted three times with hexane: chloroform (4:1, *v/v*) to obtain fatty acid methyl esters (FAME). Samples were made up with 200 µL Hexane with an internal injection standard (C19:0 methyl nonadecanoate; NuCheck Elysian, MN, USA).

FAME samples were run in accordance to AOAC official methods 963.22 (“Methyl Esters of fatty acids in oils and fats”). In brief, FAME was analyzed by gas chromatography (GC, Agilent Technologies Australia, Victoria, Australia) performed using an Agilent 6890 with an Agilent SP-2560 silica capillary column (DKSH New Zealand Limited, Auckland, New Zealand) (100 m × 0.25 mm i.d., 0.2 µm film thickness) and peak area determined by flame ionized detection (FID). Samples (1 µL) were injected via a split injector at 260 °C. The column temperature program was: 220 °C at 17 min, then raised by 2.8 °Cmin^−1^ to 240 °C and held for 5 min. Fatty acids were identified to an external commercial fatty acid standard (Supelco 37 Component FAME Mix, Merck, Auckland, NZ) using ChemStation software (Version A10.02, Agilent, Auckland, NZ). Nitrogen was the carrier gas.

### 4.6. Pharmacokinetics Calculation and Statistical Analyses

The following PK parameters were calculated for EPA, DHA, *n*-3 LC PUFA and EPA + DHA plasma concentrations out to 12 and 48 h using a non-compartmental model.

- AUC_0-t_: The area under the plasma concentration-time curve, from time t = 0 to 12 and 48 h, calculated by the linear trapezoidal method.
- C_Max_: Maximum measured plasma concentration from time 0 to 12 and 48 h.
- T_Max_: Time of the maximum measured plasma concentration to 12 and 48 h.
- t^½^: A measure of elimination, half-life is the time necessary for the concentration in the plasma to decrease by half.

If the maximum value occurred at more than one time point, T_Max_ was defined as the first time point with this value. The plasma concentrations were not baseline adjusted for the PK calculations as the concentration changes for all four analytes showed considerable variability after dosing with some participants increasing and other decreasing.

Arithmetic means and standard deviations were calculated for the AUC and C_Max_ parameters and medians and inter-quartile ranges (IQR) for T_Max_. Additionally, geometric means and 95% confidence intervals were calculated for AUC and C_Max_.

ANOVA (analysis of variance) was performed for the AUC and C_Max_ parameters to 12 and 48 h for EPA, DHA and *n*-3 LC PUFA. These parameters were ln-transformed prior to analysis. The ANOVA model included treatment and period (time) as fixed effects, and participant as a random effect. The ANOVA included calculation of least-squares means (LSM), and the differences between LSMs and the 95% confidence intervals for these differences. These differences and confidence intervals were back-transformed to produce geometric mean ratios and 95% confidence intervals.

The T_Max_ parameters were compared between treatments using Friedman’s non-parametric ANOVA with pair-wise comparisons between treatments undertaken when the Friedman’s test indicated a significant difference amongst the treatments.

A two-tailed *p*-value < 0.05 was taken to indicate statistical significance. The multiple comparison strategy used an approach called Fisher’s protected LSD, this means that only when the f-ratio from the ANOVA as significant were the pairwise tests undertaken.

## 5. Conclusions

Based on this exploratory study of EPA, DHA and *n*-3 LC PUFA pharmacokinetic parameters, we conclude: EPA peak concentrations and plasma exposures were significantly lower with GSM oil compared with GSM half-shell and GSM powder products; DHA and *n*-3 LC PUFA plasma exposure parameters were statistically comparable across the four GSM products evaluated; and GSM oil produced the shortest median time to maximal plasma *n*-3 LC PUFA concentration of all evaluated products. This was primarily due to maximal concentrations being reached during the first peak in plasma concentrations, in contrast with the other evaluated products. All products evaluated were well-tolerated, with no adverse effects assessed.

## Figures and Tables

**Table 1:** Plasma content of the pharmacokinetic parameter, area under the plasma concentration-time curve (AUC) for eicosapentaenoic acid (EPA), docosahexaenoic acid (DHA) and long chain-polyunsaturated fatty acids (*n*-3 LC-PUFA) after a single dose of frozen half-shell Greenshell™ mussel (GSM), GSM food ingredient, GSM powder and GSM oil extract with matching levels of lipid at 12 and 48 h time points. ^a,b^ Geometric means values across the column not sharing a common superscript were significantly different (*p* < 0.05) as determined by Fisher’s protected LSD (least significant difference).

	**AUC_0–12 h_**											
		AUC_0–12h_ mg/L	95.0% Lower CL for Mean	95.0% Upper CL for Mean		AUC_0–12h_ mg/L	95.0% Lower CL for Mean	95.0% Upper CL for Mean		AUC_0–12h_ mg/L	95.0% Lower CL for Mean	95.0% Upper CL for Mean
Frozen half-shell GSM	EPA	5407.0	4375.7	6681.5	DHA	8819	7457	10,429.9	*n*-3 LC PUFA	17,880.3	16,250.7	19,673.4
GSM Food ingredient		5046.2	3722.2	6841.2		8840.1	7570.1	10,323.2		18,071.9	15,091.8	21,640.5
GSM Powder		5821.7	4395.8	7710.3		7947.8	6771.1	9328.9		18,180.9	15,760.3	20,973.3
GSM Oil extract		4886.9	4137.2	5772.3		8340.8	7350.8	9464.0		17,305.3	15,516.0	19,300.8
F		−	-	-		−	-	-		−	-	-
*p*-value		−	-	-		−	-	-		−	-	-
	**AUC_0–48h_**											
		AUC_0–48h_ mg/L	95.0% Lower CL for Mean	95.0% Upper CL for Mean		AUC_0–48h_ mg/L	95.0% Lower CL for Mean	95.0% Upper CL for Mean		AUC_0–48h_ mg/L	95.0% Lower CL for Mean	95.0% Upper CL for Mean
Frozen half-shell GSM	EPA	23,963.6 ^b^	19,489.7	29,464.6	DHA	34,167.4	28,805.8	40,526.9	*n*-3 LC PUFA	73,793.4	65,863	82,678.8
GSM Food ingredient		22,138.7 ^a,b^	17,126.5	28,617.8		34,549.4	30,361.7	39,314.6		74,412.4	63,442.4	87,279.2
GSM Powder		24,683.2 ^b^	18,464.3	32,996.6		32,574.5	27,579.2	38,474.6		75,639.8	62,800.7	91,103.7
GSM Oil extract		19,858.4 ^a^	16,821.8	23,443.2		32,194.5	28,017.1	36,994.9		68,033.5	60,115.2	76,994.7
F		4.840	-	-		−	-	-		−	-	-
*p*-value		0.013	-	-		−	-	-		−	-	-

**Table 2:** Plasma content of the pharmacokinetic parameter C_Max_ (Maximum measured plasma concentration over the time span specified) for eicosapentaenoic acid (EPA), docosahexaenoic acid (DHA) and long chain-polyunsaturated fatty acids (*n*-3 LC-PUFA) after a single dose of frozen half-shell GSM, GSM food ingredient, GSM powder and GSM oil extract with matching levels of lipid for 12 and 48 h time points. ^a,b,c^ Geometric mean values across the column not sharing a common superscript were significantly different (*p* < 0.05) as determined by Fisher’s protected LSD.

	**C_Max0–12h_**											
		C_Max0–12h_ mg/L	95.0% Lower CL for Mean	95.0% Upper CL for Mean		C_Max0–12h_ mg/L	95.0% Lower CL for Mean	95.0% Upper CL for Mean		C_Max0–12h_ mg/L	95.0% Lower CL for Mean	95.0% Upper CL for Mean
Frozen half-shell GSM	EPA	546.6^c^	441.4	676.9	DHA	851	747.8	967.3	*n*-3 LC PUFA	1694	1578.5	1818.5
GSM Food ingredient		474.4 ^a,b^	359.7	625.7		834	724.1	959.3		1681	1439.6	1961.9
GSM Powder		539.0 ^b,c^	405.7	716		736	640.2	845.5		1659	1434	1918.5
GSM Oil extract		450.4 ^a^	372.1	545.1		780	711.2	855.6		1604.5	1414.2	1820.9
F		5.045	-	-		−	-	-		−	-	-
*p*-value		0.011	-	-		−	-	-		−	-	-
	**C_Max48h_**											
		C_Max0–48h_ mg/L	95.0% Lower CL for Mean	95.0% Upper CL for Mean		C_Max0–48h_ mg/L	95.0% Lower CL for Mean	95.0% Upper CL for Mean		C_Max0–48h_ mg/L	95.0% Lower CL for Mean	95.0% Upper CL for Mean
Frozen half-shell GSM	EPA	594.7 ^c^	499.8	707.5	DHA	857	759.2	968.4	*n*-3 LC PUFA	1772	1623.6	1934
GSM Food ingredient		536.8 ^a,b^	421.5	683.7		852	749.1	968		1782	1509.3	2103.4
GSM Powder		593.3 ^b,c^	445.5	790.2		835	703.6	992		1832	1504.6	2230.6
GSM Oil extract		477.4 ^a^	395.1	576.8		806	713.9	909.2		1634	1431.5	1864.7
F		6.388	-	-		−	-	-		−	-	-
*p*-value		0.004	-	-		−	-	-		−	-	-

**Table 3:** Plasma content of the pharmacokinetic parameter T_Max_ (Time of the maximum measured plasma concentration over the time span specified) for eicosapentaenoic acid (EPA), docosahexaenoic acid (DHA) and long chain-polyunsaturated fatty acids (*n*-3 LC-PUFA) after a single dose of frozen half-shell GSM, GSM food ingredient, GSM powder and GSM oil extract with matching levels of lipid for 12 and 48 h time points. ^a,b^ Arithmetic mean values across the column not sharing a common superscript were significantly different (*p* < 0.05) as determined by Friedman’s Two-Way Analysis of Variance by Ranks.

	**T_Max12h_**											
		T_Max_ (0–12 h) 25 percentile	T_Max_ (0–12 h) Median	T_Max_ (0–12 h) 75 percentile		T_Max_ (0–12 h) 25 percentile	T_Max_ (0–12 h) Median	T_Max_ (0–12 h) 75 percentile		T_Max_ (0–12 h) 25 percentile	T_Max_ (0–12 h) Median	T_Max_ (0–12 h) 75 percentile
Frozen half-shell GSM	EPA	6	6	8	DHA	2	4	6	*n*-3 LC PUFA	6	6	8
GSM Food ingredient		6	6	6		2	3	5		3	5	6
GSM Powder		5	6	7		4	4	6		4	5	7
GSM Oil extract		5	6	6		4	4	5		4	5	6
F		-	−	-		-	−	-		-	−	-
*p*-value		-	−	-		-	−	-		-	−	-
	**T_Max48h_**											
		T_Max_ (0–48 h) 25 percentile	T_Max_ (0–48 h) Median	T_Max_ (0–48 h) 75 percentile		T_Max_ (0–48 h) 25 percentile	T_Max_ (0–48 h) Median	T_Max_ (0–48 h) 75 percentile		T_Max_ (0–48 h) 25 percentile	T_Max_ (0–48 h) Median	T_Max_ (0–48 h) 75 percentile
Frozen half-shell GSM	EPA	12	24	24	DHA	2	4	6	*n*-3 LC PUFA	12	24 ^b^	48
GSM Food ingredient		24	30	30		2	4	6		15	27 ^b^	48
GSM Powder		24	24	30		4	29	48		24	30 ^b^	41
GSM Oil extract		5	15	24		4	4	18		4	5 ^a^	16
F		-	−	-		-	−	-		-	−	-
*p*-value		-	−	-		-	−	-		-	0.04	-

**Table 4:** Composition of the four samples (frozen half-shell GSM, GSM food ingredient, GSM powder and GSM oil extract) fed to clinical trial participants.

	GSM Oil Extract	Frozen Half-Shell GSM	GSM Food Ingredient	GSM Powder
Sample size (g)	2.3	125	22.5	22.5
Proximate composition	Amount per serving (g)			
Fat	2.30	2.25	2.05	2.23
Ash	0.00	1.75	4.82	3.94
Crude Protein	0.00	17.79	11.12	10.44
Carbohydrate	0.00	5.25	3.85	5.06
Fatty acid profile	Amount per serving (mg)			
C14:0 myristic acid	111.3	97.5	81.8	87.1
C16:0 palmitic acid	326.2	289.4	308.0	348.4
C16:1 palmitoleic acid	146.5	128.1	137.5	151.5
C18:0 stearic acid	84.0	88.6	69.6	92.8
C18:1n7 vaccenic acid	58.6	50.4	50.5	64.4
C18:1n9c oleic acid	48.8	21.7	24.4	24.6
C18:2n6c linoleic acid	84.0	39.5	40.0	32.2
C18:3n3 alpha linolenic acid (ALA)	25.4	26.1	27.8	17.6
C18:3n4 octadecatrienoic acid	23.4	21.0	2.6	2.5
C18:4n3 stearidonic acid (SDA)	48.8	58.0	47.0	32.2
C20:1 gadoleic acid	37.1	31.2	36.5	41.7
C20:4n6 arachidonic acid (AA)	33.2	27.4	29.6	26.5
C20:5n3 eicosapentaenoic acid (EPA)	355.5	350.0	339.4	320.0
C22:5n3 docosapentaenoic acid (DPA)	25.4	23.6	24.4	28.4
C22:6n3 docosahexaenoic acid (DHA)	267.6	311.1	174.0	189.3
∑SFA	554.1	509.8	493.4	567.8
∑MUFA	291.0	234.7	251.5	285.3
∑PUFA	879.5	880.5	718.3	684.8
∑Omega 3	727.9	776.0	622.2	595.7
∑Omega 6	140.6	91.5	77.4	69.7
Lipid class	Amount per serving (mg)			
Polar lipids (PL)	1463.6	1530.0	14.3	46.8
Sterols	29.9	104.3	43.0	82.4
Triacylglycerols (TAG)	801.9	622.5	1951.3	2051.5

∑SFA includes C15:0 pentadecanoic acid; C17:0 heptadecanoic acid; C20:0 arachidic acid and C21:0 heneicosanoic acid; ∑PUFA include C16:2n4 hexadecadienoic acid; C18:3n6 gamma linolenic (GLA); C20:3n3 cis-11, 14, 17-eicosatrienoic acid; C20:3n6 cis-8, 11, 14-eicosatrienoic acid and C20:4n3 eicosatetraenoic acid.
